# Application of chemometrics techniques to solve environmental issues in Malaysia

**DOI:** 10.1016/j.heliyon.2019.e02534

**Published:** 2019-10-04

**Authors:** Nur Zahidah Shafii, Ahmad Shakir Mohd Saudi, Jyh Chyang Pang, Izuddin Fahmy Abu, Norzahir Sapawe, Mohd Khairul Amri Kamarudin, Hammad Farhi Mohd Saudi

**Affiliations:** a Institute of Medical Science Technology, Universiti Kuala Lumpur, Kajang, Selangor, 43000, Malaysia; b Institute of Chemical and Bioengineering Technology, Universiti Kuala Lumpur, Alor Gajah, Melaka, 78000, Malaysia; c Faculty of Applied Social Sciences, Universiti Sultan Zainal Abidin, Gong Badak, Kuala Nerus, Terengganu, 21300, Malaysia; d Faculty of Economics and Muamalat, Universiti Sains Islam Malaysia, Bandar Baru Nilai, Nilai, Negeri Sembilan, 71800, Malaysia

**Keywords:** Environmental science, Chemometrics, Environment, Flood, Malaysia, Pollution, Review

## Abstract

There has been a growing concern on the rising of environmental issues in Malaysia over the last decade. Many environmental studies conducted in this country began to utilise the chemometrics techniques to overcome the limitation in the environmental monitoring studies. Chemometrics becomes an important tool in environmental fields to evaluate the relationship of various environmental variables particularly in a large and complex database. The review aimed to analyse and summarize the current evidences and limitations on the application of chemometrics techniques in the environmental studies in Malaysia. The study performed a comprehensive review of relevant scientific journals concerning on the major environmental issues in the country, published between 2013 and 2017. A total of 29 papers which focused on the environmental issues were reviewed. Available evidences suggested that chemometrics techniques have a greater accuracy, flexibility and efficiency to be applied in environmental modelling. It also reported that chemometrics techniques are more practical for cost effective and time management in sampling and monitoring purposes. However, chemometrics is relatively new in environmental field in Malaysia and various scopes need to be considered in the future as the current studies focused on very limited number of major environmental issues. Overall, chemometrics techniques have a lot of advantages in solving environmental problems. The development of chemometrics in environmental studies in the country is necessary to advance understanding, thus able to produce more significant impacts towards the effective environmental management.

## 1 Introduction

Chemometrics is known as a branch of environmental analytical chemistry that applies multivariate statistical modelling and data treatment (Simeonov et al., 2003; Otto, 2016). Chemometrics technique is one of the major tools applied in solving environmental issues as it can avoid misinterpretation of a large and complex environmental monitoring data (Simeonov et al., 2003). In the recent year, many researchers focused more on the application of chemometrics technique in determining the classification of samples and the identification of pollution sources (Levei et al., 2015; Loganathan et al., 2015; Qian et al., 2017). Current studies shown chemometrics technique becomes an important approach in environmental scopes to disclose and justify the correlation of various environmental variables (Juahir et al., 2008, 2011; Mas et al., 2010).

Environmental monitoring studies contain huge amounts of physical and chemical parameters which collected based on different geographical sites, time periods and different environmental compartments such as air, water, soil, sediments, biota and other elements (Mas et al., 2010). However, the correct methods are required for the interpretation of the large amount of data as it can be very tricky and difficult due to the complexity of data particularly in problem solving and decision making processes (Juahir et al., 2011, 2004; Azid et al., 2015a; Saudi et al., 2017). Therefore, chemometrics technique based on the multivariate statistical data analysis has been proposed as the powerful analytical tool to reveal relevant patterns and variation sources in these huge amounts of environmental databases.

Chemometrics technique involves a wide variety of methods involving parametric and non-parametric depending on the normality of the data. The increasing trend of applying chemometrics in environmental studies over the last two decades retorts to the intensive research that keen to test and validate the power of data processing techniques in environmental fields and to the availability of applicable software (Mas et al., 2010). The most common chemometrics applied in environmental studies are the hierarchical agglomerative cluster analysis (HACA or CA), discriminant analysis (DA), principal components analysis (PCA), factor analysis (FA), multiple linear regression (MLR) and artificial neural networks (ANN).

As there has been a growing concern on the rising of environmental issues in Malaysia over the last decade, many environmental studies conducted in this country began to utilise the chemometrics technique to overcome the limitation in the environmental monitoring studies. Chemometrics have been applied in various environmental scopes including air quality, air pollution, water quality, water pollution, flood pattern, land use changes, sedimentation and erosion (Azid et al., 2014a, 2015a; Saudi et al., 2014a, Saudi et al., 2015a, 2017; Kamaruddin et al., 2015; Isiyaka and Juahir, 2015; Ismail et al., 2016; Rwoo et al., 2016; Samsudin et al., 2017a). From these studies, application of chemometrics offered many great impacts towards more effective environmental management in the country.

The aim of this review was to analyse the application of chemometrics technique concerning the environmental issues specifically in Malaysia. In addition, this current review also aimed to determine the effectiveness of chemometrics based on the current evidences from previous environmental studies in the country over the last few years.

## 2 Main text

### 2.1 Materials and methods

The review was conducted as a systematic process using computer-based electronic search to access all journals related to the application of chemometric technique in Malaysia, published between 2013 and 2017. The study began by collecting information from established and high impact scientific publications using internet search engine such as Google Scholar and Researchgate, and variety of the following search terms, either separately or combined: *chemometrics, environment, air pollution, air pollutant index, air quality, water quality, flood, cluster analysis, discriminant analysis, multi linear regression, principal component analysis, factor analysis, artificial neural network, time series analysis, pattern recognition,* and *Malaysia*.

All primary research of any designs published between 2013 and 2017, which explored the assessment and analysis on the environmental issues in Malaysia using any chemometrics approach were included in the review. A total of 51 studies were successfully collected. After removal of duplicates, the papers were initially reviewed by abstract before proceed to final assessment to increase the credibility of the review. The final assessment of the review was based on the inclusion and exclusion criteria as stated below. Overall, a total of 29 studies were selected for a more detail review.

**Fig. 1:**
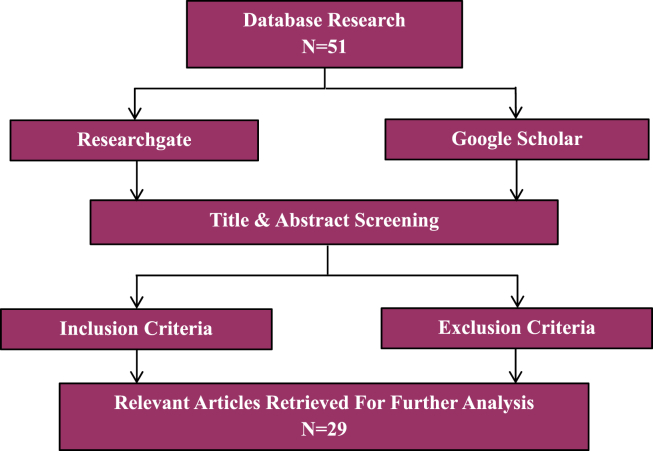
Research strategy for the review.

The inclusion criteria includes (1) All selected environmental studies set in Malaysia, (2) Primary studies describing application of chemometrics, (3) All research designs, (4) Publication between January 1, 2013, and December 31, 2017, and (5) All in the English language. While, the exclusion criteria includes (1) All studies had not been cited and (2) Editorials, commentaries and reviews. The research strategy used in the review is illustrated in Fig. 1 below.

### 2.2 Results

The selected publications were reviewed with the aim of showing the evolution of chemometrics applications that was being representative by the different data sets. Most of the studies had similarity in study design but varied by different chemometric methods, scopes of the study and geographic region of Malaysia. Table 1 shows the classification of different chemometrics techniques employed in the studies reviewed in this paper based on the different data structure and aims of the study.

**Table 1:** Classification of different chemometrics employed in the reviewed articles.

Chemometrics techniques	
**Unsupervised learning methods** • Cluster analysis (CA) • Artificial neural network (ANN)	**Correlation and regression analysis** • Multiple linear regression (MLR)
**Supervised learning methods** • Discriminant analysis (DA) • Artificial neural network (ANN)	**Time series analysis** • Statistical process control (SPC)
**Factorial methods** • Factor analysis (FA) • Principal component analysis (PCA)	

According to 29 selected papers, the studies mainly focused on three main environmental issues in Malaysia which were: (1) air quality and pollution (n = 11), (2) water quality and pollution (n = 12) and (3) flood (n = 6) as summarized in Tables 2, 3 and 4, respectively.

**Table 2:** Application of chemometrics technique in air quality and pollution.

No.	Research aims	Chemometrics technique	Key findings	References
1.	Identification of spatial and temporal air quality pattern recognition at three selected Malaysian air monitoring stations based on 7 years database (January 2000–December 2010).	DA, HACA, PCA and ANN	The usefulness of chemometrics and ANN modelling techniques in evaluating and interpreting large air quality datasets in order to enable to get better information about the air quality pattern.	Mutalib et al. (2013)
2.	Application of Feed-Forward Artificial Neural Network Model to predict the API within the seven selected Malaysian air monitoring stations in the southern region of Peninsular Malaysia based on 7 years database (2005–2011).	PCA and ANN	Rotated PC scores are more efficient and effective in reducing the predictor variables without losing important information. The ANN method can be applied successfully as tools for decision making and problem solving for better atmospheric management.	Azid et al. (2013)
3.	Prediction of the level of air pollution using Principal Component Analysis and Artificial Neural Network techniques at ten monitoring stations in Malaysia for 7 years (2005–2011)	PCA and ANN	A significant reduction of input data using rotated PCA scores with a good predictive power similar to the standard model. ANN models are good alternatives air pollution modelling.	Azid et al. (2014a)
4.	Identification of spatial variations on API at 8 monitoring stations in the southern region of peninsular Malaysia for 3 years (2005–2007).	HACA, DA, PCA-FA, ANN and MLR	ANN model shows better prediction compared to the MLR. An excellent exploratory tool in API assessment, identification and apportionment of pollution sources, and interpretation of complex dataset.	Azid et al. (2014b)
5.	Identification of potential source apportionment of air pollution in ten Malaysian monitoring stations for 7 years (2006–2012).	PCA	Application of the PCA method can be applied for the source apportionment purpose for the future and effective management of air quality.	Azid et al. (2015c)
6.	Assessment of the spatial variation and source apportionment of air pollution at five selected monitoring stations in Peninsular Malaysia covering 2007–2011 (5years).	HACA, PCA and MLR	Cut down the cost of equipment, reducing cost and time of monitoring redundant stations and pollutants.	Isiyaka et al. (2015)
7.	Recognition of the pollution sources and identification the most significant pollutant at 14 monitoring stations around Peninsular Malaysia from January to December 2007.	HACA, DA, PCA-FA and MLR	Provide meaningful information on the spatial variability of a large and complex air quality data. Practical and cost-effective for a new air quality monitoring network.	Azid et al. (2015a)
8.	Selection of the most significant variables of air pollutants using sensitivity analysis at ten selected Malaysian monitoring stations based on database for a 7 year period (2006–2012).	ANN-API-AP, ANN-API-LCO, ANN-API-LO_3_, ANN-API-LPM_10_, ANN-API-LSO_2_, ANN-API-LNO_2_, ANN-API-LCH_4_, ANN-API-LNmHC, ANN-API-LTHC, ANN-API-LO, and ANN-API-DOE	ANN-API-LO model was the best predictor model as only two parameters utilized as input for API prediction. API prediction with the use of fewer parameters has been highly practicable for air quality management due to its time and cost efficiency.	Azid et al. (2015b)
9.	Identification of the spatial variation of air pollutants and its pattern at seven air monitoring stations in the northern part of Peninsular Malaysia for 4 years (2008–2011).	HACA, DA and ANN	The predictive ability of chemometrics is at least as good as the standard model. The feed-forward ANN model could predict API values from all existing input with slight precision and could save time and cost of monitoring purposes.	Amran et al. (2015)
10.	Determination of air quality pattern at Putrajaya monitoring station based on three years observation (2011–2013).	PCA, FA and SPC	PCA and FA model identified five pollutants that affected air quality. SPC analysis verified that SO_2_ was the main pollutant.	Kamaruzzaman et al. (2017)
11.	Assessment of ambient air pollution pattern in Shah Alam, Malaysia for 5 years (2009–2013).	PCA and SPC	The level of air quality was manipulated by the climate condition, gas, non-gas and secondary air pollutants.	Zakaria et al. (2017)

ANN = Artificial Neural Network; API = air-pollutant index; AP = all parameters; DA = discriminant analysis; DOE = Department of Environment; FA = factor analysis; HACA = hierarchical agglomerative cluster analysis; LCO = leave carbon monoxide; LO_3_ = leave ground-level ozone; LPM_10_ = leave particulate matter; LSO_2_ = leave sulphur dioxide; LNO_2_ = leave nitrogen dioxide; LCH_4_ = leave methane; LNmHC = leave non-methane hydrocarbon; LTHC = leave total hydrocarbon; LO = leave-out; MLR = multiple linear regression; PCA = principal components analysis; SO_2_ = sulphur dioxide; SPC = statistical process analysis.

**Table 3:** Application of chemometrics technique in water quality and pollution.

No.	Research aims	Chemometrics technique	Key findings	References
1.	Identification of the surface water pollution at 13 monitoring stations of Terengganu River Basin from 5 years (2003–2007)	CA, DA and PCA-FA	The usefulness for analysis and interpretation of complex databases. The basis for the spatial variations and prospective of pollution sources.	Ibrahim et al. (2015)
2.	Analysis the relationship between the physicochemical levels and the drinking water quality in water treatment plant at Kuala Kubu Bharu, Malaysia (January to December 2011).	DA and PCA-FA	The drinking water quality was within the national standards. The ideal way into provide meaningful information on the spatial variability of a large and multifaceted drinking water quality data.	Rwoo et al. (2014)
3.	Assessment of water quality from 50 selected monitoring stations of Johor River for 5 years (2003–2007).	HACA, DA and PCA	One monitoring station of each cluster is sufficient to represent a reasonably accurate spatial water quality assessment for the entire river, thus reduce the needs for numerous sampling stations.	Dali et al. (2014)
4.	Spatial assessment of water quality affected by the land-use changes in the Kuantan River Basin for 6 years (2003–2008).	MLR, HACA, DA and PCA	A good and efficient prediction of missing data using MLR model. The use of chemometric techniques for analysing and interpreting complex water quality data.	Saudi et al. (2014a)
5.	Classification of water quality at nine monitoring stations in the Muda River Basin for 10 years data set (1998–2007)	CA, PCA and DA	Chemometric techniques were effective for river water classification as it exhibited a correct classification efficiency of 100%.	Azhar et al. (2015)
6.	Analysis of surface water pollution in the eight monitoring sites along Kinta River for 8 years (2006–2013).	CA, DA, PCA and MLR	A reduction in the number of monitoring stations and parameters for a cost effective and time management in the monitoring processes. A simple explanation for a proper policy implementation by government and stakeholders involve in water quality management.	Isiyaka and Juahir (2015)
7.	Determination of water quality status for six monitoring stations at Linggi River based on 6 years observation (1997–2012).	CA and PCA	Reliability of surface water classification facilitate local authorities to reduce cost of monitoring cost by reducing the monitoring station and guide for better decision making.	Kasim et al. (2015)
8.	Spatial characterization of water quality data and identification of pollutants sources from 12 sampling stations at Terengganu River for 5 years (2006–2010).	HACA and PCA	The application disclosed important information on the spatial variability of large and complex river water quality data sets to control pollution sources.	Kamaruddin et al. (2015)
9.	Variation of physicochemical sources for drinking water quality at 28 water treatment plants in Klang Valley for a 4 year period (2009–2012).	DA and PCA	The significant input on the spatial variability of a large and multifaceted drinking water quality data. A reference in the future for other related studies.	Rwoo et al. (2016)
10.	Determination of the spatial variation and source identification of heavy metal pollution in 8 monitoring stations along the Straits of Malacca for 5 years (2006–2010).	CA, DA and PCA	Monitoring of heavy metals could be optimal through a single state, each representing the northern and southern region. A time saving, cost effective regime of heavy metal monitoring should be designed by the related authorities to ensure continuous observation of these pollutants.	Ismail et al. (2016)
11.	Assessment of river water quality assessment at 39 monitoring stations at Johor River Basin for 5 years (2003–2007).	PCA, APCS-MLR and SPC	The most significant parameters which contributed to the river pollution should be used as reference for authority as it could save time and money budget in water quality sampling and lab analysis of the redundant parameters.	Samsudin et al. (2017a)
12.	Detection of control limit for source apportionment in 11 monitoring stations Perlis River Basin for 5 years (2003–2007).	PCA, APCS-MLR and SPC	The significant parameters that impacted the water quality could be used as a reference by the government agencies for monitoring purposes.	Samsudin et al. (2017b)

ANN = Artificial Neural Network; APCS = absolute principal component scores; CA = cluster analysis; DA = discriminant analysis; HACA = hierarchical agglomerative cluster analysis; FA = factor analysis; MLR = multiple linear regression; PCA = principal components analysis; SPC = statistical process analysis.

**Table 4:** Application of chemometrics technique in flood.

No.	Research aims	Chemometrics technique	Key findings	References
1.	Identification and recognition of flood risk pattern in four monitoring stations at Kuantan River Basin for 30 years (1982–2012).	FA, Time series analysis and ANN	The prediction of the risk class for flood would construct proper mitigating measure more efficiently for flood occurrence, thus able to cut down cost of destruction and save life.	Saudi et al. (2014b)
2.	Recognition of flood risk pattern in four monitoring stations at Muda River Basin for 30 years (1982–2012).	FA, Time series analysis and ANN	The prediction was accurate and could be used for future prediction in the risk assessment for flood occurrence. Development of earlier warning system for flood prevention.	Saudi et al. (2014c)
3.	Assessment of flood risk index in four monitoring stations at Johor River Basin for 30 years (1982–2012).	FA, SPC, ANN and FRI	Water level was the most practicable variable to be used for the warning alert system. The current and future prediction of flood risk index model shown to be efficient.	Saudi et al. (2015a)
4.	Prediction of hydrological modelling for flood risk in four monitoring stations at Langat River Basin covered a 30 year period (1982–2012).	ANN, FA and SPC	The prediction of risk in Risk Class was accurate and relevant to be taken into consideration for future flood prediction.	Saudi et al. (2015b)
5.	Development of new flood risk index in tropical area, Muda River Basin for 30 years (1982–2012).	PCA, SPC, ANN and FRI	The control limit of water level allowed a continuous monitoring system by local authorities to execute early preventive measures. The significant and accurate prediction could be used for future prediction in the risk assessment for flood occurrence. SPC was practical to be used for the drought conditions.	Saudi et al. (2017)
6.	Analysis of the relationship of rainfall pattern and water level on major flood in Pahang River Basin in 2014.	PCA and SPC	High relationship between rainfall and water level. Hydrological pattern and trend were extremely affected by climate. Importance of flood assessment and forecasting in the future.	Sulaiman et al. (2017)

ANN = artificial neural network; DA = discriminant analysis; FA = factor analysis; FRI = flood risk index; HACA = hierarchical agglomerative cluster analysis; MLR = multiple linear regression; PCA = principal components analysis; SPC = statistical process analysis.

#### 2.2.1 Air quality and pollution

A study was conducted in Malaysia, which focused on the prediction of the air pollution level using PCA and ANN techniques (Table 2) (Azid et al., 2014a). PCA was used to identify the potential sources of variations in air quality. The combination of PCA-ANN was developed to implement the integration of data-driven modelling in predicting selected Malaysian Air Pollutant Index (API). The model was a useful tool in air pollution modelling as it showed better predictive ability in the determination of API with fewer variables. The study had similar findings with others studies that concluded the chemometrics technique could be a simple and efficient alternative model to provide reliable estimation of API not only with limited information but with a large and complex database (Azid et al., 2013, 2014b; Mutalib et al., 2013; Amran et al., 2015).

Another study in Peninsular Malaysia was aimed on the spatial air quality modelling using chemometrics techniques, which include HACA, DA, PCA-FA and MLR (Azid et al., 2015a). The main goals of the study were to recognize the pollution sources and to identify the most significant pollutant influenced the air quality in the study area. The findings from HACA and DA indicated that a better monitoring network approach could be proposed which might lessen the quantity of monitoring stations. The study recommended that a new air quality monitoring network should be designed in term of practical and cost-effective for a better and effective air quality management. These findings were also been supported by studies conducted by Azid et al. (2015c), Isiyaka et al. (2015) and Azid et al., 2015b, Azid et al., 2015c.

Kamaruzzaman et al. (2017) and Zakaria et al. (2017) show the application of chemometrics technique specifically PCA and statistical process control (SPC) model were significant to identify the sources of air pollutants and the pattern of air pollution in Putrajaya and Shah Alam, Malaysia. The efficacy of these studies had supported the previous study by Azid et al. (2014b) for determining the identification and apportionment of pollution sources from a complex air quality database.

#### 2.2.2 Water quality and pollution

A chemometric study as shown in Table 3 was performed on the spatial characterization and identification sources of pollution at Terengganu River Basin (Kamaruddin et al., 2015). The study focused on determining the possible main contributor of water pollution by implementing multivariate analysis on the water quality data obtained from 12 sampling stations at the study area. The study revealed that chemometric technique could disclose useful and important information for the local authorities on the spatial variability of a large and complex river water quality data in order to effectively control pollution sources as well as for future research references. The findings of the study were similar to Saudi et al. (2014a), Ibrahim et al. (2015), Azhar et al. (2015) and Samsudin et al. (2017b).

Meanwhile, Isiyaka and Juahir (2015) studied on surface water pollution in Kinta River using chemometrics techniques. CA and DA were used to determine the spatial characteristics in the similarities of water quality monitoring processes in the Kinta River which resulted in drastic reduction in the number of monitoring stations and observed parameters. PCA was applied to identify the most statistical significant parameters and possible sources of water pollution in the river. The study showed that anthropogenic activities and natural processes constituted the major possible sources of pollution. The study proposed a reduction in the number of monitoring stations and parameters for a cost effective and time management in water quality monitoring processes, parallel to Dali et al. (2014) and Kasim et al. (2015) studies.

Moreover, a study was conducted on spatial assessment and source identification of heavy metals pollution in surface water along the Straits of Malacca using several chemometric techniques (Ismail et al., 2016). The study aimed to identify heavy metal pollution distribution and the most significant sources via a comprehensive application of multivariate analyses including CA, DA and PCA, on data sets obtained from the Department of Environment (DOE). CA indicated that that one state from each region is sufficient in representing the heavy metal contamination of marine systems in surface water along the Straits of Malacca. The findings further confirmed by DA which showed discriminating variables in the spatial variance of the heavy metals. The study concluded that the monitoring of heavy metals could be optimally performed via a single state, each representing the northern and southern region of the Straits of Malacca which would save time and reduce cost of monitoring.

A case study conducted at water treatment plants in Klang Valley focused on the assessment of the variation of physicochemical sources for drinking water quality using DA and PCA techniques (Rwoo et al., 2016). The purposes of the study were to analyse the physiochemical activities and heavy metals activities in drinking water samples collected from the 28 treatment plants, and to detect the source of pollution for the most revealing parameters. It was suggested that this modelling could be used as reference in the future studies to ensure more priority efforts should be taken promptly through laws and legislations enforcement in controlling point and non-point pollution sources for an effective management of the Malaysian drinking water quality. In addition, the quality of drinking water should meet the requirements of national standard in ensuring the cleanliness of the water to Malaysians (Rwoo et al., 2014).

A recent study was carried out in Johor on river water quality assessment using the goodness of the receptor modelling approach, absolute principal component scores (APCS)-MLR and SPC methods (Samsudin et al., 2017a). The study focused on determining the most significant parameters of each river basin which contribute to river pollution loading, discovering the potential contamination of pollutants and performing the process capability of water quality. The study proposed that continuous monitoring should be done by DOE to ensure that the level of ammonia-nitrogen (NH_3_–N) and phosphate (PO_4_) concentration in Johor river basin complies with the specification limit of the National Water Quality Standard (NWQS).

#### 2.2.3 Flood

One study carried out in Kuantan River Basin focused on the flood risk pattern recognition using chemometric technique of FA, Time Series Analysis and ANN (Table 4) (Saudi et al., 2014b). The aims of this study were to identify the main flood contributor and to predict hydrological modelling as well as the risk of flood occurrence at the study area. This study recommended that proper mitigation measure can be executed more efficiently for Kuantan community before, during and after flood occurrence, thus reducing the impacts towards physical destruction and life. Strict action by local government on the development along the river must be enforced accordingly to the Environmental Quality Act (EQA) 1974. Also, a study in the Pahang River Basin by Sulaiman et al. (2017) shown there is a relationship between the rainfall and water level during the big floods in Pahang in 2014. Hydrological patterns during the disaster were also influenced by climate change and weather. Therefore, assessment and prediction of flood are strongly emphasized for future impacts.

Furthermore, a flood risk index assessment study in Johor River Basin was conducted to determine the factor of flood occurrence, to set up the control limit for flood, to evaluate the accuracy of flood risk prediction and to predict the future control limit for flood in the river basin (Saudi et al., 2015a). The chemometric methods involved FA, SPC, ANN and Flood Risk Index (FRI) model. This study highlighted that water level is the most practicable variable to be used for the warning alert system as it have been proven statistically to accentuate on the flood pattern visualization and optimal rates for maximum limit for flood control in Johor. The formation of efficient control limits which is sensitive to changes in water level can enable flood warning alerts to improve the existing system used by Department of Irrigation and Drainage Malaysia (DID) in managing, controlling and preventing flood in Malaysia (Saudi et al., 2014c). The comparison between the current and future prediction of flood risk index shown the risk model used in this study was generally efficient to bring major changes to the global flood control issues (Saudi et al., 2014b, 2014c; 2015a).

Another flood study emphasized the application of SPC method on the new flood risk index in tropical region in Malaysia (Saudi et al., 2017). The study focused on determining the main cause of flood occurrence, flood risk pattern, and also the prediction performance of new FRI created for flood risk control in the study area. The control limit system obtained from SPC method allowed local authorities to conduct a continuous monitoring system and able to execute emergency response plan as early as in cautionary zone risk class level in order to avoid massive destruction occurs. The study also suggested that SPC method was also practical to be used in preparation for the drought conditions in Malaysia.

### 2.3 Discussion

#### 2.3.1 The advantages of chemometric techniques

**Fig. 2:**
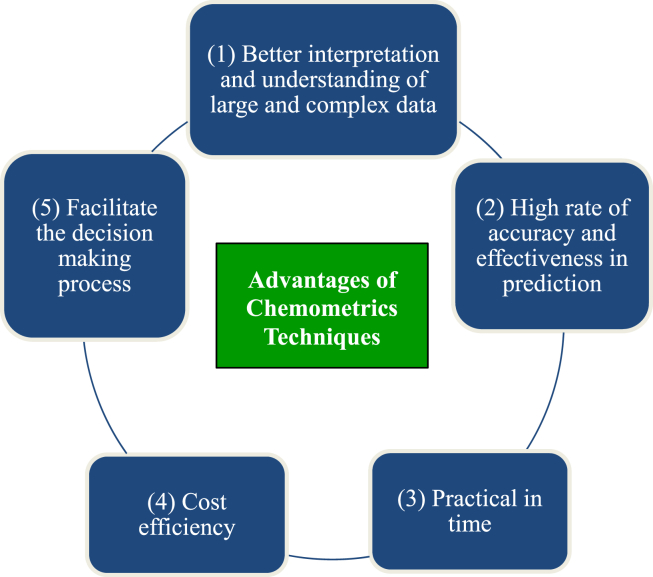
The advantages of the chemometrics.

The review from the selected studies provides information and evidences on the advantages of chemometric techniques applied in the environmental studies in Malaysia (Fig. 2). In conjunction with the findings of the current review, it was found that chemometric techniques succeed in lessening the complexity of large amounts environmental monitoring data. The review proves the previous studies that shown the application of integrated chemometric technique help in the interpretation of a set of complex data for a better understanding in solving environmental problems (Massart and Kaufman, 1983; Simeonov et al., 2003; Kannel et al., 2007). The application of the variety of chemometric techniques on environmental data reveals significant information based on the spatial and temporal variability of environmental data that can be used by the local authorities and stakeholders to implement effective prevention and control measures (Juahir et al., 2011; Mutalib et al., 2013; Isiyaka et al., 2014).

The review also reported that chemometric techniques are more practical for theirs cost and time efficiency in sampling and monitoring purposes. The techniques successfully disclose meaningful information from complex datasets by determining only the optimum number of sampling parameters or stations needed to be used as the input for the environmental modelling (Saudi et al., 2014a; Azid et al., 2015a, 2015b). Reliability of the techniques is very beneficial to the local authorities to focus more on the significant parameters or area of monitoring that influenced by the pollution and these will help them to save time and expenses in sampling, and also avoid the redundant parameters (Simeonov et al., 2003; Kasim et al., 2015; Ismail et al., 2016; Samsudin et al., 2017a). Besides, the difficulties and limitations on the quantitative chemical analysis of organic pollutants in environmental samples which involves expensive and time consuming laboratory analysis indeed make chemometrics more appealing and better approach to complement analytical methodologies (Mas et al., 2010).

The available evidences from the review suggested that chemometric techniques have a greater accuracy, flexibility and efficiency to be applied in environmental modelling. Identification of the limitations for all parameters developed from SPC analysis can assist local authorities to implement effective mitigation measures to control environmental pollutions and natural disasters like flood and drought. The application of ANN particularly for pattern classification, prediction and optimization had been proven in solving environmental related problems (Kapageridis, 2002). The current review shown that almost all studies utilised ANN have high rate of accuracy for prediction (more than 90%) with the lowest error which implied the best model for predictive performance. Moreover, the risk models developed based on a historical data proved that the effectiveness of the same model to be used for future prediction in environmental risk assessment (Samsudin et al., 2017a, Samsudin et al., 2017b; Saudi et al., 2017). The development of risk models is also important in disaster management, for instance, in flood alert warning system and emergency response plan based on the risk level from the prediction (Saudi et al., 2015a). Thus, chemometric techniques present as the best tool in decision making process and problem solving for environmental management (Azid et al., 2013; Isiyaka et al., 2014, 2015).

#### 2.3.2 Implications towards environmental management in Malaysia

The impacts of application of chemometric techniques focused on the three main environmental issues in Malaysia, which are air, water and flood. Ambient air quality is monitored by the Department of Environment (DOE) Malaysia and it is reported based on the Malaysian Air Pollutant Index (API), a non-dimensional number system calculated based on the daily average concentrated of major air pollutants (Isiyaka et al., 2015). The department currently has 52 Continuous Air Quality Monitoring (CAQM) stations and 14 Manual Air Quality Monitoring (MAQM) stations nationwide and the State Government recently adopts 6 air pollutants as opposed to previously 5 air pollutants; consist of carbon monoxide (CO), nitrogen dioxide (NO_2_), sulphur dioxide (SO_2_), ozone (O_3_), particulate matter 10 micrometres or less in diameter (PM_10_) and a new pollutant of particulate matter 2.5 micrometres or less in diameter (PM_2.5_) (DOE, 2019). Azid et al. (2014b) proposed that the chemometric techniques and modelling is an excellent tool in API assessment as it has better predictive ability in determination of API with fewer sampling parameters and stations thus can be setbacks in designing a novelty air quality monitoring network particularly in controlling point and non-point pollution sources.

Similarly, Malaysian DOE also responsible to monitor water quality and water pollution for both river and marine. A 2016 report by DOE stated that a total of 1,064 manual stations in 146 river basins were monitored under Manual Water Quality Monitoring (MWQM) Programme and 10 automatic monitoring stations for Continuous Water Quality Monitoring (CWQM) Programme. River water quality status is determined by the Water Quality Index (WQI) and the National Water Quality Standards for Malaysia (NWQS). WQI derived based on sub-index of 6 main parameters include dissolved oxygen (DO), biochemical oxygen demand (BOD), chemical oxygen demand (COD), nitrogen ammonia (NH_3_–N), total suspended solids (TSS) and pH, to indicate the level of water pollution. And, NWQS that contained 72 parameters is used for classification of river. The report revealed the river water quality had been declining while the polluted river was increased in 2016 compared in 2015 (DOE, 2016). Mamun and Zainudin (2013) recommended that WQI and NWQS to be reviewed to improve their consistency and compatibility. This was also addressed by a previous study and it confirmed that multivariate analysis is effective for river water classification (Azhar et al., 2015). In addition, the significantly parameters that influenced the river water quality can be served as references by DOE for monitoring purposes (Samsudin et al., 2017a).

A new National Flood Forecasting and Warning System (NaFFWS/PRAB) has been developed by Malaysian Department of Irrigation and Drainage (DID) aims to provide accurate and reliable forecast and warning of impending floods for public safety and to reduce the impact of hazard and disaster risks (DID, 2018). NaFFWS/PRAB is an integrated system that utilised hydrodynamic modelling and technology application which able to forecast flood 7 days earlier and warning dissemination in 2 days before the flood (DID, 2018). The components of the NaFFWS are fully automated systems driven by a combination of live, telemetered gauged data from DID's own InfoBanjir database, spatial rainfall radar data, and numerical weather prediction rainfall forecasts from the Malaysian Meteorological Department (HR Wallington, 2018). However, current evidences of the flood risk model developed from chemometric techniques emphasized water level is the best variable for flood warning alert system as monsoon season and rainfall were not significant factors for flood occurrence (Saudi et al., 2014b, 2014c, 2017). An efficient control limits that sensitive to water level changes can assist and improve the existing flood warning system used the DID (Saudi et al., 2015a, 2015b). Besides, significant variables that affect water level can be used as guideline by local authorities to implement strict enforcement of laws and regulations towards developers in controlling the excessive amount of surface runoff into the river (Saudi et al., 2014b, 2015a).

#### 2.3.3 Limitations

Chemometrics is widely used in analytical chemistry since 30 years ago with the first publication in this research area in the 1980s (Malinowski and Howery, 1980; Massart and Kaufman, 1983; Mas et al., 2010). Afterward, chemometric techniques gradually applied in other disciplines including environmental sciences by scientists and engineers (Einax et al., 1998; Kowalkowski et al., 2005; Fianko et al., 2009). Application of chemometrics in Malaysia for environmental solving problems only significantly revolved in the earliest 2010s particularly for water quality assessment (Juahir et al., 2004, 2011), followed by air quality and pollution (Dominick et al., 2012; Azid et al., 2013), and flood risk pattern (Saudi et al., 2014b). There are little environmental studies on the application of chemometric techniques in Malaysia have been published. The main reason for this limitation mainly due to chemometrics is relatively new in environmental field in the country. Moreover, the various scopes of the study need to be taken into consideration as the current studies focused on limited number of environmental issues.

## 3 Conclusion

The application of chemometrics techniques demonstrated that it is a useful and effective tool in solving environmental issues in Malaysia as there are a lot of advantages towards environmental management have been identified in the study. However, this current review has shown that the further development of the chemometrics itself is necessary to advance understanding, thus able to present more significant impacts towards future and better management of environment particularly in Malaysia. Active collaboration locally or globally among researchers, local authorities, stakeholders and industrials potentially bring major changes for the environment in the long run.

## Declarations

### Author contribution statement

All authors listed have significantly contributed to the development and the writing of this article.

### Funding statement

This work was supported by the Universiti Kuala Lumpur for the Short Term Research Grant (STRG).

### Competing interest statement

The authors declare no conflict of interest.

### Additional information

No additional information is available for this paper.
